# *In Vitro* Drug Susceptibility of a *Leishmania (Leishmania) infantum* Isolate from a Visceral Leishmaniasis Pediatric Patient after Multiple Relapses

**DOI:** 10.3390/tropicalmed8070354

**Published:** 2023-07-04

**Authors:** Bianca A. Ferreira, Gustavo de A. Santos, Elizabeth M. Coser, Juliana M. Sousa, Mônica E. A. Gama, Leônidas L. B. Júnior, Fabrício S. Pessoa, Mayara I. S. Lima, Silvia R. B. Uliana, Adriano C. Coelho

**Affiliations:** 1 Departamento de Biologia Animal, Instituto de Biologia, Universidade Estadual de Campinas (UNICAMP), Rua Monteiro Lobato, 255, Campinas 13083-862, Brazilelizabethmcoser@hotmail.com (E.M.C.); 2 Departamento de Biologia, Centro de Ciências Biológicas e da Saúde, Universidade Federal do Maranhão, São Luís 65080-805, Brazil; 3 Programa de Pós-Graduação em Saúde e Ambiente, Centro de Ciências Biológicas e da Saúde, Universidade Federal do Maranhão, São Luís 65080-805, Brazil; 4 Departamento de Medicina, Centro de Ciências Biológicas e da Saúde, Universidade Federal do Maranhão, São Luís 65080-805, Brazil; 5 Hospital Universitário, Universidade Federal do Maranhão, São Luís 65080-805, Brazil; 6 Departamento de Parasitologia, Instituto de Ciências Biomédicas, Universidade de São Paulo, São Paulo 05508-000, Brazil

**Keywords:** *Leishmania (Leishmania) infantum*, visceral leishmaniasis, drug susceptibility

## Abstract

The parasitic protozoan *Leishmania (Leishmania) infantum* is the etiological agent of human visceral leishmaniasis in South America, an infectious disease associated with malnutrition, anemia, and hepatosplenomegaly. In Brazil alone, around 2700 cases are reported each year. Treatment failure can occur as a result of drug, host, and/or parasite-related factors. Here, we isolated a *Leishmania* species from a pediatric patient with visceral leishmaniasis that did not respond to chemotherapy, experiencing a total of nine therapeutic relapses and undergoing a splenectomy. The parasite was confirmed as *L. (L.) infantum* after sequencing of the ribosomal DNA internal transcribed spacer, and the clinical isolate, in both promastigote and amastigote forms, was submitted to *in vitro* susceptibility assays with all the drugs currently used in the chemotherapy of leishmaniasis. The isolate was susceptible to meglumine antimoniate, amphotericin B, pentamidine, miltefosine, and paromomycin, similarly to another strain of this species that had previously been characterized. These findings indicate that the multiples relapses observed in this pediatric patient were not due to a decrease in the drug susceptibility of this isolate; therefore, immunophysiological aspects of the patient should be further investigated to understand the basis of treatment failure in this case.

## 1. Introduction

The parasitic protozoan *Leishmania (Leishmania) infantum* is the etiological agent of human visceral leishmaniasis (VL) in South America, the Mediterranean Basin, the Middle East, and Central Asia, and is transmitted to humans and other mammals through the bite of infected sand flies. VL is the main clinical form of leishmaniasis that affects the phagocytic mononuclear system of the liver, spleen, and/or bone marrow, and may be fatal if untreated in 90% of cases [1,2]. The disease is considered an opportunistic infection in patients coinfected with human immunodeficiency virus (HIV) and in patients on immunosuppressive or immunomodulatory treatments for organ transplantation [3]. More than 90% of VL cases in Latin America occur in Brazil, and, from 2018 to 2020, around 2700 cases were reported each year, with cases occurring in almost all Brazilian states, although most were concentrated in the northeast and north regions [2]. Children up to 5 years old and immunocompromised adults are the major risk groups for VL [1]. The main clinical manifestations of VL are persistent fever, hepatosplenomegaly, and pancytopenia accompanied by hypergammaglobulinemia and weight loss [1,4]. In endemic areas of Brazil, seroprevalence of antibodies to *L. (L.) infantum* can be as high as 34.9% in children [5,6]. In pediatric patients, risk factors for developing the clinical disease include immaturity of the immune response, immunosuppressive diseases, and malnutrition [7].

Patients with VL in Brazil are mainly treated with meglumine antimoniate (SbV), amphotericin B (AmB) deoxycholate, or liposomal AmB (L-AmB), with cure rates of at least 80% [3,8]. For L-AmB in particular, cure rates of around 87% have been reported for this clinical form of the disease [8]. Although the antileishmanial drug miltefosine (MF) is not approved for the treatment of VL in Brazil, it has proven to be highly effective in the treatment of VL in patients infected with *L. (L.) donovani* in India, with cure rates higher than 90% [9,10]. In a clinical trial in Brazil, the effectiveness of MF in patients with VL was only 60%, with a treatment failure of approximately 52% in pediatric patients and 26% in adults [11,12]. A deletion in chromosome 31 of *L. (L.) infantum* isolates obtained from patients in this trial was identified and correlated with an increased risk of MF treatment failure [11]. This genomic region contains four genes, 3′-nucleotidase/nucleases (*NUC1* and *NUC2*), helicase-like protein, and 3,2-trans-enoyl-CoA isomerase, and is known as the miltefosine sensitive *locus* (MSL) [11,12]. The absence of *NUC1* and *NUC2* genes was later shown to be specifically responsible for the reduction of MF susceptibility in parasites, due to a higher resistance to lipid metabolism perturbations caused by the drug [13].

Treatment failure in leishmaniasis may be related to drug-, host-, and/or parasite-related factors [14]. Certain genetic characteristics or the immune status of the patient, such as HIV coinfection or non-HIV-related immunocompromised states, are the main host-related factors associated with treatment failure in VL [4,15]. On the other hand, the main parasite-related factor that may lead to treatment failure is drug resistance, which may be intrinsic and/or acquired [14,16]. Intrinsic resistance, also known as natural resistance, is a molecular/biochemical feature that makes the parasite tolerant to the drug, while acquired resistance is a phenotype selected after exposure of the parasite to the drug used during treatment [16].

Here, we characterized the *in vitro* drug susceptibility of a *L. (L.) infantum* isolate from a pediatric patient who presented multiple VL relapses, despite undergoing appropriate treatment regimens with SbV, L-AmB, and MF [17]. Evidence of primary or secondary immunodeficiencies was not found after seven relapses [17], and the patient underwent a splenectomy owing to the persistence of hypersplenism and a clinical condition compatible with the active disease. After a total of nine relapses, parasites were collected from a bone marrow aspirate to evaluate the *in vitro* susceptibility to standard antileishmanial drugs.

## 2. Materials and Methods

### 2.1. Patient Description and Ethics Statement

A 10-year-old male patient from Itapecuru city, State of Maranhão, Brazil, a highly endemic region for VL, was initially diagnosed with VL when he was 3 years old. The patient had experienced a total of nine relapses since the first episode occurred in August 2016 [17]. In the last relapse, the patient presented hepatomegaly, a liver palpable at 10 cm below the costal margin, pancytopenia (3350 white blood cells/μL; 6.1 g/dL hemoglobin; and 89,000 platelets/μL), and a myelogram positive for *Leishmania* species. Bone marrow aspirates were collected immediately before starting a new treatment scheme with a combination of L-AmB at 3 mg/kg/day intravenously for 15 days (totaling 50 mg/kg), pentamidine (PEN) at 4 mg/kg/day for 15 days (totaling 60 mg/kg), and MF at 50 mg/day orally for 28 days. The Research Ethics Committee of the Universidade Federal do Maranhão (protocol ID 3.921.086) approved the procedures involving the collection of bone-marrow-aspirate samples from the patient, and this procedure was conducted after receiving the parents’ consent.

### 2.2. Isolation of the L. (L.) infantum ME Isolate, Parasite Cultivation and Animals

Bone-marrow-aspirate samples were subjected to initial cultivation in Schneider’s medium (Sigma-Aldrich, St. Louis, MO, USA) supplemented with 20% heat-inactivated fetal bovine serum (FBS; Thermo Scientific), 10 U/mL penicillin, and 10 μg/mL streptomycin, incubated at 25 °C. After isolation, promastigotes of the clinical isolate (MHOM/BR/2021/ME) and of the *L. (L.) infantum* strain (MHOM/BR/1972/LD) were grown at 25 °C in M199 medium (Sigma-Aldrich) supplemented with 40 mM HEPES (pH 7.4), 0.1 mM adenine, 0.005% hemin, 10% FBS, 10 U/mL penicillin, 10 μg/mL streptomycin, and 2% sterile male human urine [18,19]. To type the clinical isolate as *L. (L.) infantum*, genomic DNA was isolated using DNAzol (Thermo Scientific), and typing was performed through PCR amplification followed by sequencing of the internal transcribed ribosomal (ITS) DNA as previously described [20]. The GenBank accession number of the ITS sequence of the isolate, referred to as the ME isolate, is ON804484.

Bone-marrow-derived macrophages (BMDMs) were obtained from BALB/c mice, aged 4–6 weeks, which were differentiated and cultured in RPMI 1640 medium (Thermo Scientific) supplemented with 10% FBS, 0.1 M sodium pyruvate, and 100 μg/mL penicillin/streptomycin and incubated in a 5% CO_2_ atmosphere at 37 °C, as previously described [21].

### 2.3. In Vitro Drug Susceptibility of Promastigotes and Intracellular Amastigotes

Drugs used in the present study were AmB deoxycholate, trivalent antimoniate (SbIII), PEN, MF (Sigma-Aldrich), SbV (Sanofi-Aventis), and paromomycin sulfate (PM; Gold Biotechnology). All drugs were diluted in Milli-Q ultrapure water to give stock solutions of 10–100 mM, which were then filter-sterilized (0.22 μm pore size) and stored at −20 °C until use, with the exception of AmB deoxycholate, which was diluted in DMSO (Sigma-Aldrich).

The drug susceptibility of the ME isolate and the LD strain promastigotes was determined using the MTT [3-(4,5-dimethyl-2-thiazolyl)-2,5-diphenyl-2H-tetrazolium bromide] colorimetric assay after 2 × 10^6^ parasites per well in 96-well plates were incubated for 24 h with the serially diluted (1:2) drugs: 200 to 3.12 nM for AmB deoxycholate; 1000 to 15.6 μM for SbIII; 25 to 0.39 μM for PEN; 200 to 3.12 μM for MF; and 800 to 12.5 μM for PM, as previously described [22]. At least three independent experiments were performed in triplicate. The optical density was determined in a plate reader (Multiskan Sky, Thermo Scientific) using a reference wavelength of 690 nm and a test wavelength of 595 nm. The 50% effective concentration (EC_50_) was determined from a sigmoidal regression of the concentration-response curves generated in GraphPad Prism 7.

For the drug susceptibility of the intracellular amastigotes, BMDMs were first plated at a density of 3 × 10^5^ macrophages per well in complete RPMI 1640 medium on round glass coverslips in 24-well plates and incubated in a 5% CO_2_ atmosphere at 37 °C for 24 h. Following this, macrophages were infected with stationary-phase promastigotes of the ME isolate and the LD strain at a ratio of 20:1 (parasites:macrophage) and incubated in a 5% CO_2_ atmosphere at 37 °C. After 3–4 h, non-internalized parasites were removed by washing with warmed phosphate-buffered saline (PBS), and the drugs in RPMI 1640 were added: AmB deoxycholate serially diluted (1:2) from 50 to 0.78 nM; MF serially diluted (1:2) from 20 to 0.31 μM; PM at 0.4, 0.2, 0.1, 0.075, 0.05, 0.025, and 0.01 μM; and PEN at 200, 100, 50, 10, 1, 0.5, and 0.1 μM. Infected macrophages were maintained for 72 h in a 5% CO_2_ atmosphere at 37 °C. SbV susceptibility assays with amastigotes were performed using drug concentrations of 1000, 750, 500, 250, 100, 50, and 25 μM, and infections were incubated for 6 days, with a replacement of medium containing SbV after 3 days. Macrophages were fixed in methanol (Sigma-Aldrich), and the Panoptic hematological method (Laborclin, Brazil) was used to stain the cells. The percentage of infection and the number of amastigotes per macrophage were determined by counting 100 macrophages in three independent experiments, which were subsequently used to determine the EC_50_ values.

All drug susceptibility assays were performed within ten *in vitro* passages for the ME isolate. Statistical significance was determined using the Student’s *t*-test in GraphPad Prism 7. Significance was considered as *p* < 0.05.

### 2.4. PCR for the Presence/Absence of Miltefosine Sensitive Locus (MSL)

To confirm the presence or absence of the MSL in *L. (L.) infantum* ME isolate, a previously described PCR protocol was used [11]. As control for the presence of the MSL genes, genomic DNA of *L. (L.) donovani* DD8 strain was used in PCR reactions. Primers targeting the genes *LinJ.31.2370* and *LinJ.31.2400* that encode the 3′-nucleotidase/nuclease (NUC1) and 3,2-transenoyl-CoA isomerase, respectively, were used, as these genes are located inside the MSL. These PCR protocols amplify fragments of 1.42 kb and 1.56 kb, respectively. As a positive control for the PCR reaction, a protocol targeting the *hsp70* gene was performed, which amplifies a fragment of 1.28 kb [23]. The primers and PCR conditions used for the amplification of the MSL and *LinJ.31.2370, LinJ.31.2400,* and *hsp70* genes were previously described [11,23].

## 3. Results and Discussion

After a total of nine therapeutic VL relapses, bone marrow aspirates were collected from the patient for parasite isolation and confirmation of the *Leishmania* species. The full ITS nucleotide sequence of the ME clinical isolate displayed at least 99.52% identity with the ITS of other *L. (L.) infantum* strains and isolates deposited in GenBank, and 99.91% identity with the *L. (L.) infantum* LD strain.

To determine whether the relapses were due to drug resistance, we evaluated the *in vitro* susceptibility of both the promastigote and amastigote forms of the parasite to various antileishmanial drugs, SbV, AmB deoxycholate, and MF, to which the patient had previously been exposed, and PEN and PM, two drugs that the patient had not been previously exposed to. The EC_50_ values for all drugs were determined in parallel with the LD strain that was originally isolated from a patient with VL in the Amazon region, and which has been used as a laboratory strain for several years [24,25]. This strain is considered to be susceptible to all these drugs.

Firstly, the ME isolate presented a similar pattern of promastigote growth *in vitro* as the LD strain (data not shown). In the susceptibility assays, the promastigote form of the ME isolate had a similar sensitivity to all tested drugs (AmB deoxycholate, PEN, PM, MF, and SbIII) as the LD strain (Table 1).

**Figure 1:**
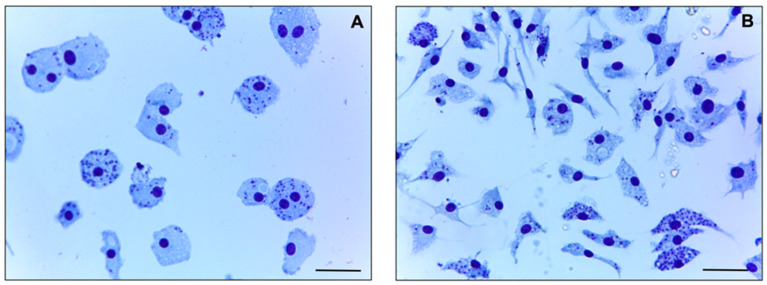
*In vitro* infection of (**A**) *L. (L.) infantum* LD strain and (**B**) the ME clinical isolate. BMDMs were infected with stationary-phase promastigotes at 37 °C and 5% CO_2_ on coverslips in 24-well plates. After 3 h, the wells were washed to remove non-internalized parasites, and the plate was incubated further under the same conditions. After 72 h, infected macrophages were fixed in methanol (Sigma-Aldrich), stained by the Panoptic hematological method (Laborclin, Brazil), then visualized on a light microscope. Bar: 10 μm.

The ME isolate and the LD strain also had similar rates of infection in BMDMs, with 69 ± 4% and 62 ± 6% infection rates, respectively; in addition, the number of amastigotes per macrophage was 5.39 ± 1.45 for the ME isolate and 6.41 ± 0.86 for the LD strain (Figure 1). The amastigote form of the ME isolate was similarly susceptible to SbV, AmB deoxycholate, and MF as the LD strain, with no significant difference in the EC_50_ values (Table 1), despite the patient being refractory to these treatments [17]. Differently, laboratory-selected SbV- and AmB-resistant *L. (L.) infantum* and *L. (L.) donovani* lines exhibit a minimum of a 3-fold increase in EC_50_ values, reaching more than a 10-fold increase when compared with their corresponding parental strains [28,29,30,31,32]. Finally, the LD strain and the ME isolate were also uniformly susceptible to PEN and PM (Table 1).

**Figure 2:**
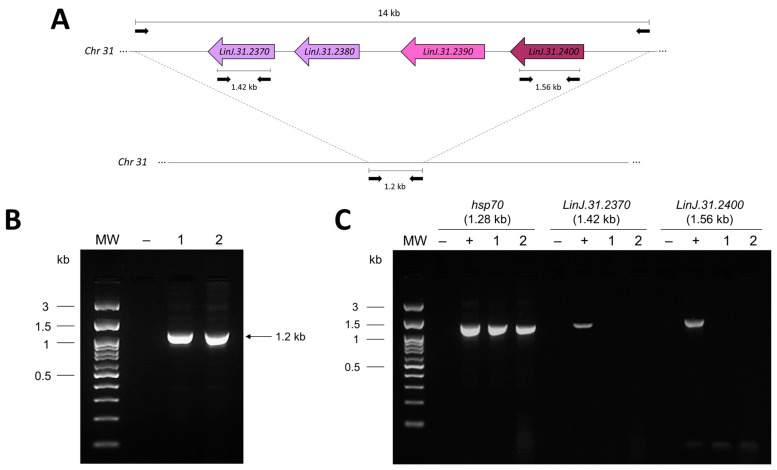
Investigation of the MSL in the *L. (L.) infantum* LD strain and ME clinical isolate, using PCR protocols previously described by Carnielli et al. [11]. (**A**) Schematic representation of the MSL of approximately 14 kb. In the absence of the MSL, a 1.2 kb DNA fragment is amplified. (**B**) PCR for the presence (~14 kb) or absence (~1.2 kb) of the MSL in the LD strain (lane 1) and the ME isolate (lane 2). (**C**) PCR amplification of the MSL genes *LinJ.31.2370* (that encodes *NUC1*) and *LinJ.31.2400* (that encodes 3,2-trans-enoyl-CoA isomerase). The size of amplified PCR products is indicated above the figure. As a control, all genomic DNAs were also evaluated by a PCR protocol that amplifies a 1.28 kb product of the *hsp70* gene, as previously described by Montalvo et al. [23]. MW—molecular weight in kilobase (kb); (−) negative control for PCR (absence of genomic DNA); (+) positive control for PCR (genomic DNA of *L. (L.) donovani* DD8 strain); (1) *L. (L.) infantum* LD strain; (2) *L. (L.) infantum* ME clinical isolate.

An increased risk of MF treatment failure in VL caused by *L. (L.) infantum* was recently associated with a deletion in chromosome 31 in this species [11]. Considering that the pediatric patient presented a relapse after MF treatment using 50 mg/day orally for 28 days, we investigated whether the MSL was also absent in the ME isolate. The absence of the MSL is demonstrated by the presence of a 1.2 kb PCR-amplified fragment, rather than a 14 kb PCR product corresponding to the complete MSL (Figure 2A). This 1.2 kb fragment was amplified from the ME clinical isolate, indicating that the MSL had been deleted (Figure 2B) [11]. The MSL was also absent in the LD strain (Figure 2B), as previously described [22]. The absence of the MSL in the ME isolate was confirmed by PCR targeting two genes located within this *locus*, *LinJ.31.2370* and *LinJ.31.2400*, that code for NUC1 and 3,2-trans-enoyl-CoA isomerase, respectively (Figure 2A). No amplification for these genes was observed in the ME isolate and the LD strain, while the expected fragments were detected for the *L. (L.) donovani* DD8 strain (Figure 2C), which contains the MSL, as previously described [22]. Brazilian *L. (L.) infantum* isolates with the MSL deletion were recently identified across the country [33]. Although the patient experienced MF treatment failure and the ME isolate was lacking the MSL, this isolate was highly susceptible to MF *in vitro* and thus does not corroborate the previously reported association between the absence of the MSL and low susceptibility *in vitro* [12]. Similar findings were described by Espada et al. [34] who, after the evaluation of almost 50 *L. (L.) infantum* isolates, did not find a correlation between the deletion of the MSL and MF resistance *in vitro*.

After parasite isolation, the patient received a course of treatment using a combination of L-AmB at 3 mg/kg/day intravenously for 15 days (totaling 50 mg/kg), PEN at 4 mg/kg/day for 15 days (totaling 60 mg/kg), and MF at 50 mg/day orally for 28 days. No toxicity related to the treatment was observed, and the patient responded well, with clinical improvement and no other episode of relapse reported after 2 years of follow-up. Importantly, this patient underwent a splenectomy after the seventh relapse [17], a procedure that may be useful for the reduction of parasite burden in refractory cases and cases of treatment failure [35,36]. The spleen is one of the most important organs of the reticuloendothelial system, being considered the main parasite reservoir in VL [1]. The successful use of a triple-combination drug therapy (L-AmB, PEN, and SbV) has already been reported in a 1-year-old child, after no clinical response to two courses of L-AmB treatment [37].

The parasite isolated after multiple courses of treatment in the pediatric patient did not appear to have acquired resistance to any antileishmanial drug. Our findings indicate that the treatment failure is probably due to genetic and/or immunophysiological aspects of the patient, which should be further investigated.

## Figures and Tables

**Table 1:** *In vitro* activity of antileishmanial drugs against promastigotes and intracellular amastigotes of the *L. (L.) infantum* LD strain and the ME clinical isolate.

Drugs	Promastigotes ^c^		Intracellular Amastigotes ^c^		CC_50_ ^d^
	LD	ME	LD	ME	
AmB ^a^	19.83 ± 4.39	26.34 ± 3.87	5.17 ± 0.95	8.84 ± 2.32	127.36 ± 0.94
SbV ^b^	-	-	269.40 ± 7.23	314.93 ± 5.84	>2000
SbIII ^b^	131.06 ± 2.60	163.10 ± 2.06	-	-	-
MF ^b^	16.15 ± 1.36	13.28 ± 1.65	1.53 ± 0.45	1.46 ± 0.21	49.52 ± 2.93
PEN ^b^	4.04 ± 1.12	2.47 ± 0.28	0.11 ± 0.01	0.10 ± 0.013	0.36 ± 0.02
PM ^b^	78.71 ± 3.15	74.28 ± 4.45	1.71 ± 0.62	2.05 ± 0.60	536.60 ± 27.1

^a^ Concentrations in nM; ^b^ Concentrations in µM; ^c^ EC_50_ mean values ± standard deviation; ^d^ Toxicity on BMDM. CC_50_ values for AmB, MF, SbV, and PM were previously reported [22,26,27]. CC_50_ for PEN was determined as described by Coser et al. [26]. (-) Data not determined.

## Data Availability

All data presented in this study are available upon request. The ITS sequence generated in this study has been deposited in GenBank under accession number ON804484.

## Abbreviations

(L-)AmB, (liposomal) amphotericin B; BMDM, bone-marrow-derived macrophages; HIV, human immunodeficiency virus; ITS, ribosomal DNA internal transcribed spacer; MF, miltefosine; MSL, miltefosine sensitive *locus*; PEN, pentamidine; PM, paromomycin; SbIII, trivalent antimoniate; SbV, meglumine antimoniate; VL, visceral leishmaniasis.
